# Prognostic value of pretreatment neutrophil-to-lymphocyte and platelet-to-lymphocyte ratios in multiple myeloma patients treated with thalidomide-based regimen

**DOI:** 10.1007/s00277-020-04092-5

**Published:** 2020-05-26

**Authors:** Aneta Szudy-Szczyrek, Radosław Mlak, Michał Mielnik, Michał Szczyrek, Aleksandra Nowaczyńska, Iwona Homa-Mlak, Szymon Zmorzyński, Kinga Kuśmierczuk, Jacek Sompor, Agata Filip, Teresa Małecka-Massalska, Marek Hus

**Affiliations:** 1 Chair and Department of Haematooncology and Bone Marrow Transplantation, Medical University of Lublin, 20-081 Lublin, Poland; 2 Chair and Department of Human Physiology, Medical University of Lublin, 20-080 Lublin, Poland; 3 Chair and Department of Pneumology, Oncology and Allergology, Medical University of Lublin, 20-090 Lublin, Poland; 4 Department of Cancer Genetics with Cytogenetics Laboratory, Medical University of Lublin, 20-080 Lublin, Poland; 5 Department of Traumatology and Emergency Medicine, Medical University of Lublin, 20-081 Lublin, Poland

**Keywords:** NLR, PLR, Prognostic factors, Multiple myeloma, Thalidomide

## Abstract

Neutrophils to lymphocytes ratio (NLR) and platelets to lymphocytes ratio (PLR) are considered as laboratory markers of inflammation. They can be potentially useful in predicting the course of multiple neoplasms including selected hematological cancers. The aim of the study was to assess the value of NLR and PLR in predicting the effects of therapy and prognosis in multiple myeloma patients treated with thalidomide-based regimen. The study group consisted of 100 patients treated with the first line CTD (cyclophosphamide, thalidomide, and dexamethasone) chemotherapy. The NLR and PLR were calculated before treatment. High NLR was observed in patients with higher stage of the disease, with poor performance status, hypercalcemia, and high CRP. High PLR was associated with low BMI and high CRP. In patients with high NLR, significantly shorter PFS was observed (17 vs. 26 months, *p* = 0.0405). In addition, high values of NLR and PLR were associated with significantly shorter OS (38 vs. 79 months, *p* = 0.0010; 40 vs. 78 months, *p* = 0.0058). Summarizing, NLR and PLR have a significant independent prognostic value for multiple myeloma patients. Furthermore, the NLR can be a predictive marker for the outcome of thalidomide-based chemotherapy.

## Introduction

Multiple myeloma (MM) is the second most common hematological neoplasm, characterized by the accumulation of malignant plasma cells in the bone marrow leading to anemia, bone pain, renal impairment, hypercalcemia, and infections.

It accounts for about 1% of all malignancies and about 10% of hematologic malignancies. It most often occurs in people in the 7th and 8th decade of life, significantly more often in men [1]. The advances made in the treatment of multiple myeloma in the last few decades, starting from the use of autologous hematopoietic stem cell transplantation, followed by the introduction of innovative therapies based on immunomodulatory drugs and proteasome inhibitors, radically improved the prognosis in this group of patients. According to current statistics, the percentage of 5-year survival is currently 48.5% and the median overall survival (OS) exceeded 6 years. Unfortunately, MM is still considered to be an incurable disease [2].

Traditional, classic prognostic factors for multiple myeloma patients include the stage of the disease, performance status, age, and comorbidities. There is a high interest in a number of factors, both genetic, biochemical and hematological, and their potential use as prognostic markers [3, 4]. Markers of inflammation are particularly interesting. It is believed that they can indirectly reflect the status of the bone marrow microenvironment, which affects the processes of regulation and promotion of growth, survival, migration, and even drug resistance of myeloma cells [5].

The aim of the study was to assess the prognostic and predictive value of NLR and PLR ratios calculated on the basis of the absolute number of neutrophils, lymphocytes, and platelets in patients with multiple myeloma treated with thalidomide-based induction chemotherapy.

## Materials and methods

The study group consisted of 100 multiple myeloma (MM) patients aged 53–69 years (median 64). All patients received triplet CTD induction therapy in 28-day cycles, in the following doses: thalidomide 100 mg/day p.o., cyclophosphamide 300–500 mg/week p.o., and dexamethasone 10–20 mg/day p.o. on days 1–4 and 8–11. The median of cycles of chemotherapy was 6. The median follow-up was 41.5 months. Demographic and clinical data including sex, age, stage, and type of disease were collected. An analysis of classic cytogenetic and biochemical prognostic factors (deletion of 17p, *t*(4;14) translocation, *t*(14;16) translocation, β2 microglobulin, LDH, CRP), NLR, and PLR (calculated as ratios of absolute neutrophil count to lymphocyte and platelet counts before treatment) was performed. In publications on the importance of NLR and PLR in assessing the prognosis of solid tumors, authors provide various cut-off points for NLR and PLR. To find the optimal values, we analyzed the ROC curves, which allowed to set the cut-off points: 2.86 for NLR and 157.66 for PLR. Data regarding treatment, such as the number of chemotherapy cycles, type of response, progression-free survival (PFS), and overall survival (OS), were also documented.

Statistical analysis of the obtained data was carried out using MedCalc 15.8 (MedCalc Software, Belgium) and Statistica 10 (Statsoft, USA) computer software. The comparison of the values of selected laboratory markers, demographic, and clinical factors was carried out using the non-parametric *U* Mann-Whitney test. The correlation between selected demographic, clinical and laboratory factors, as well as NLR and PLR was carried out using Spearman’s rank correlation. The analysis of ROC curves was used to determine the cut-off points for NLR and PLR. The Kaplan-Meier estimation method and Cox logistic regression were used to assess the probability of survival and the occurrence of progression depending on the distribution of the studied factors. In all used tests, results with *p* values < 0.05 were considered statistically significant.

## Results

### Influence of demographic and clinical factors on studied laboratory parameters

**Fig. 1:**
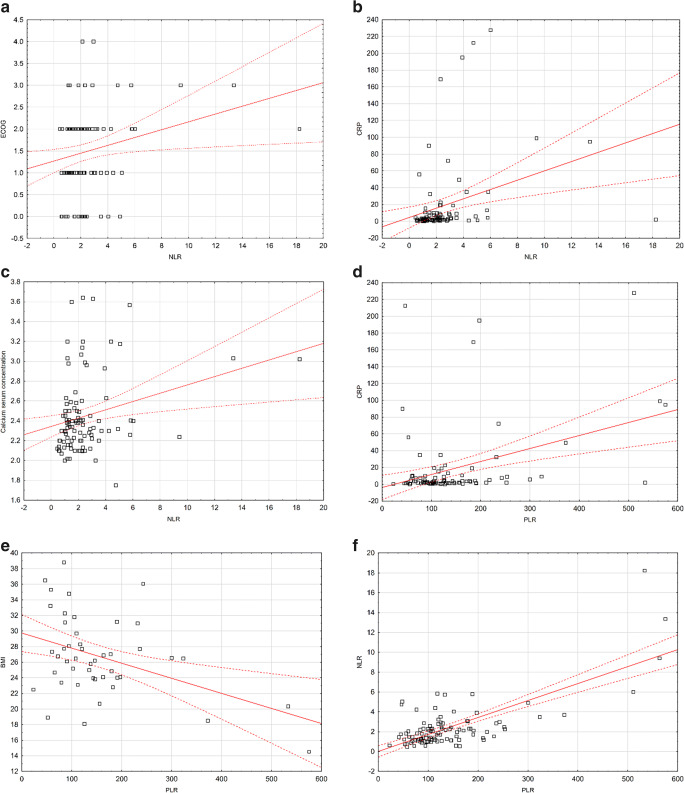
**a** Spearman’s correlation between performance status and NLR. **b** Spearman’s correlation between CRP serum concentration and NLR. **c** Spearman’s correlation between calcium serum concentration and NLR. **d** Spearman’s correlation between CRP and PLR. **e** Spearman’s correlation between BMI and PLR. **f** Spearman’s correlation between PLR and NLR

**Fig. 2:**
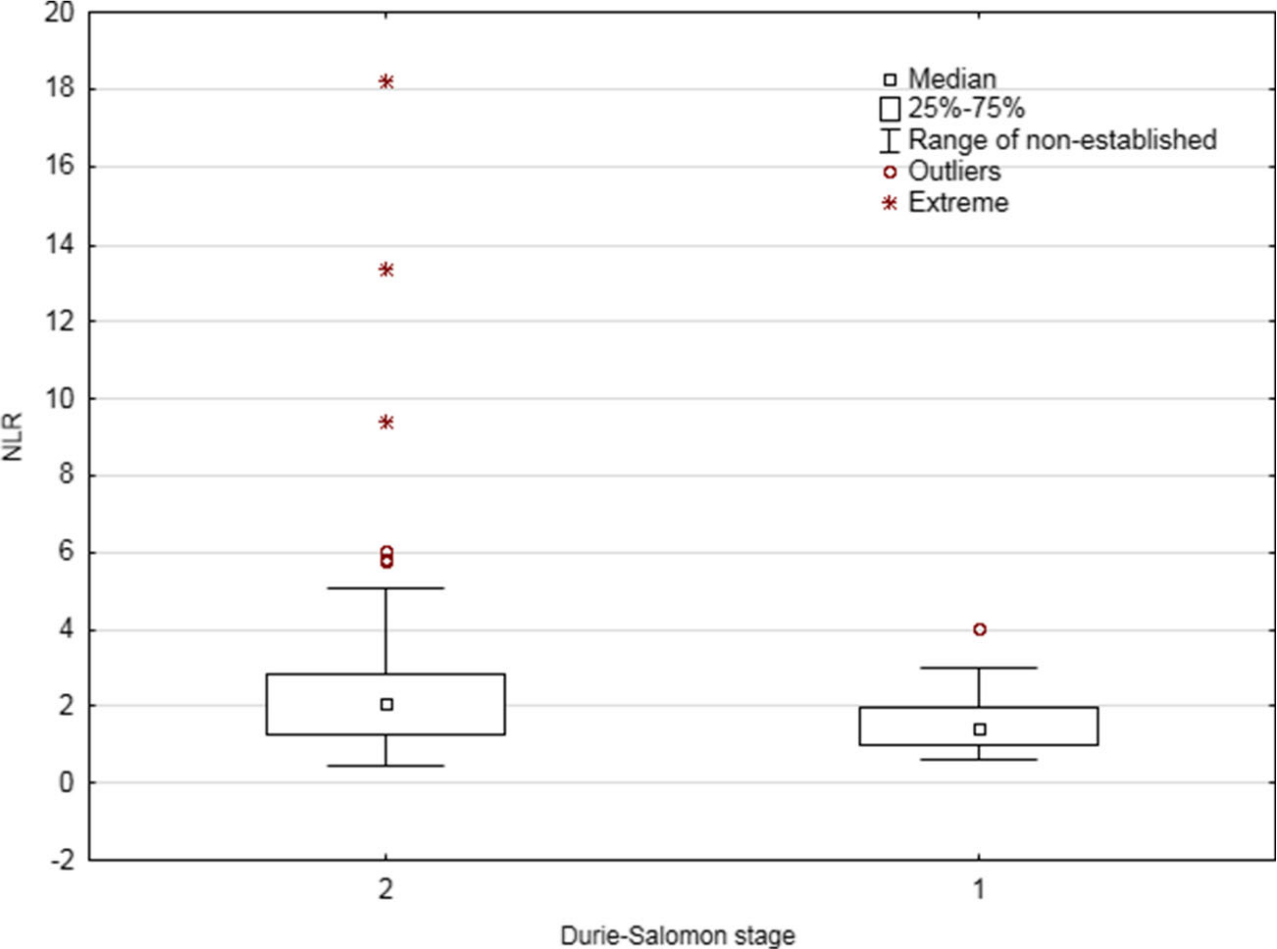
Comparison of NLR values depending on the level of advancement according to Durie-Salomon criteria

The distribution of the values of the examined laboratory parameters (NLR, PLR) was not significantly correlated with factors such as sex, age, type of the disease (secretory or non-secretory MM), type of monoclonal protein, class of light chains, percentage of plasma cells in bone morrow, and specific cytogenetic abnormalities: (del(17p), *t*(4:14), and *t*(4:16)) (Fig. 1). Among the studied factors, only disease stage according to Durie-Salomon scale was significantly related to the NLR index. Statistically significantly higher NLR values were observed in patients at a higher Durie-Salomon stage (III vs. I or II: 2.06 vs. 1.43, *p* = 0.0404; Fig. 2). Detailed information on the comparison of the values of selected laboratory indicators depending on demographic and clinical factors are included in the Table 1.

**Table 1:** Comparison of the values of selected laboratory indicators depending on demographic and clinical factors

Variable	NLR	*p*	PLR	*p*
	*Me*		*Me*	
Sex				
Men	1.79	0.3903	122.74	0.6200
Women	2.03		111.63	
Age				
< 60	1.92	0.6987	119.20	0.5320
> 60	1.93		115.45	
Diagnosis				
Secretory (1)	1.97	0.6696	119.40	0.4116
Light chain disease (2)	1.86		109.28	
Non-secretory/plasmablastic (3)	1.07		68.39	
Non-secretory/plasmacytoma (4)	2.20		190.35	
Secretory. Light chain disease				
Non-secretory: plasmablastic	1.92	0.7449	117.44	0.8466
plasmacytoma	1.96		137.30	
Monoclonal protein class				
IgA	2.73	0.0587	126.02	0.3892
IgG	1.73		111.64	
Light chain type				
Kappa	1.76	0.4602	109.20	0.5581
Lambda	2.20		118.46	
Durie-Salmon stage				
I	1.51	0.2282	122.77	0.9456
II	1.38		114.08	
III	2.04		118.06	
I, II	1.43	0.0404	106.59	0.5727
III	2.06		118.26	
I	1.51	0.3567	122.77	0.7385
II, III	1.97		117.44	
ISS stage				
1	2.04	0.7824	126.90	0.4770
2	1.71		107.76	
3	1.80		111.89	
1	2.04	0.5566	126.90	0.2396
2, 3	1.78		109.82	
1,2	2.00	0.9775	120.49	0.3852
3	1.80		111.89	
Deletion 17p				
Absent	1.98	0.3004	114.06	0.9075
Present	1.32		120.61	
Translocation t(4;14)				
Absent	1.98	0.5287	114.06	0.8828
Present	1.38		125.14	
Translocation t(4;16)				
Absent	1.83	–	120.61	–
Present	0.75		57.03	
Renal function				
A	1.92	0.3062	119.40	0.4326
B	2.07		105.22	
Stage of chronic kidney disease	1.97	0.6186	113.63	0.5978
G1	1.54		137.58	
G2	1.76		120.34	
G3a	2.32		146.00	
G3b	1.84		114.06	
G4	4.74		46.58	
G5D	1.49		93.39	
G1	1.97	0.3406	118.46	0.6207
G2, G3a, G3b, G4, G5D	1.84		114.08	
Performance status				
0	2.04	0.1244	140.44	0.5000
1	1.48		107.25	
2	1.98		118.46	
3	2.33		127.39	
4	2.53		112.72	
0,1	1.70	0.0682	115.45	0.5125
2–4	2.17		119.40	
Body weight loss before treatment				
No	1.80	0.0876	107.76	0.2367
Yes	2.12		122.74	
5%	1.68	0.1344	107.49	0.2040
10%	2.31		127.27	
Anemia grade before treatment (WHO)				
Absent	1.84	0.5366	154.38	0.5630
I (mild)	2.12		185.23	
II (moderate)	1.76		163.14	
III (severe)	2.20		130.77	
IV (life-threatening)	1.31		162.10	
Absent	1.84	0.3920	119.21	0.9633
I (mild), II (moderate), III (severe), IV (life-threatening)	2.06		115.45	

However, we noted a statistically significant weak positive correlation between the performance status and NLR (rho = 0.197, *p* = 0.0492; Fig. 1a). In addition, this indicator correlated positively with serum calcium concentration and CRP (weak, rho = 0.223, *p* = 0.0258, moderate, rho = 0.449, *p* < 0.0001; respectively; Fig. 1b, c). Furthermore, PLR also correlated positively with CRP (weak, rho = 0.225, *p* = 0.0311; Fig. 1d). This indicator showed a negative correlation with BMI (rho = − 0.300, *p* = 0.0429; Fig. 1e). There was also a moderate, positive correlation between NLR and PLR (rho = 0.453, *p* = 0.0091; Fig. 1f). Spearman’s correlations are shown in the Table 2.

**Table 2:** Spearman’s correlation between selected demographic-clinical and laboratory factors and NLR and PLR

Variable	NLR		PLR	
	rho	*p*	rho	*p*
Age	− 0.060	0.5520	− 0.166	0.0995
Durie-Salmon stage	0.171	0.0899	0.024	0.8142
ISS stage	− 0.025	0.8022	− 0.114	0.2592
Performance status	0.197	0.0492	0.006	0.9519
Percentage of plasmocytes	− 0.027	0.7941	− 0.196	0.0514
Anemia grade before treatment (WHO)	0.037	0.7133	− 0.105	0.2970
HGB	− 0.061	0.5483	0.077	0.4443
eGFR	− 0.037	0.7137	0.029	0.7711
Stage of chronic kidney disease	0.064	0.5287	− 0.016	0.8781
ALB	− 0.082	0.4200	− 0.056	0.5774
CREA	0.144	0.1516	− 0.078	0.4421
ALP	− 0.048	0.7009	0.017	0.8917
B2M	− 0.035	0.7327	− 0.143	0.1610
LDH	− 0.020	0.8569	− 0.148	0.1741
Calcium	0.223	0.0258	0.154	0.1249
CRP	0.449	< 0.0001	0.225	0.0311
Time to auto-HSCT	− 0.073	0.6164	0.025	0.8662
BMI	− 0.183	0.2237	− 0.300	0.0429
NLR and PLR	0.453, 0.0091			

### Response

**Fig. 3:**
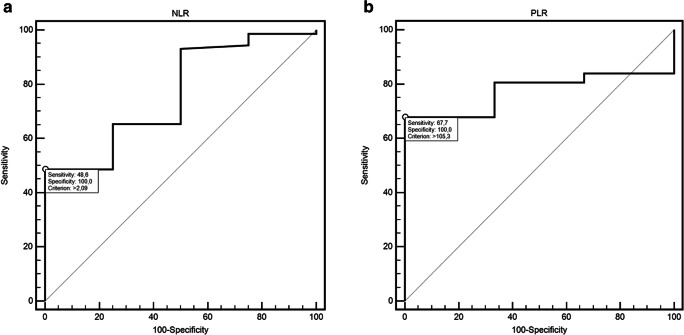
**a** ROC curve illustrating response detection after 6-th cycle of CTH according to NLR value. **b** ROC curve illustrating response detection after 8-th cycle of CTH according to PLR value

There were no statistically significant differences between studied laboratory indicators: NLR and PLR and the type of clinical response obtained during thalidomide-based chemotherapy (CTH) cycles (Tables 1 and 2). However, ROC curve analysis (Table 3) showed that NLR might be useful in predicting response after the 6th cycle of CTH (48.6% sensitivity and 100% specificity; AUC = 0.76, 95% CI 0.65–0.85; *p* = 0.0303; Fig. 3a). On the other hand, PLR showed 94.4% sensitivity and 50% specificity in detection of response after 8th cycle of CTH (AUC = 0.77, 95% CI 0.56–0.90; *p* = 0.0011; Fig. 3b).

**Table 3:** The influence of selected factors on the risk of PFS and OS shortening in multivariate analysis (Cox logistic regression model) (model adjustment for PFS: *p* < 0.0001; χ^2^ = 41.40 and OS: *p* < 0.0001; χ^2^ = 44.24)

Variable	Progression free survival				Overall survival			
	β	*p*	HR	95% CI	β	*p*	HR	95% CI
Sex								
Male	− 0.17	0.6935	0.84	0.35–1.99	2.56	0.0012	12.88	2.75–60.34
Age								
≥ 65	0.80	0.1090	2.22	0.84–5.86	0.81	0.1331	2.24	0.79–6.39
Translocation t(4;14)								
Present	2.72	< 0.0001	15.18	4.98–46.26	1.35	0.0218	3.84	1.22–12.07
Auto HSCT								
No	0.10	0.8333	1.11	0.43–2.82	0.98	0.0745	2.65	0.91–7.71
Stage of chronic kidney disease								
G3a/G3b/G4/G5D	− 1.28	0.0864	0.28	0.06–1.19	1.47	0.1016	4.35	0.75–25.03
Weight loss before treatment								
Yes	0.75	0.2039	3.34	1.06–10.53	2.43	0.0001	11.39	3.50–37.08
ALB								
Low	1.24	0.0021	3.47	1.58–7.64	1.19	0.0235	3.28	1.18–9.11
Creatinine								
High	1.75	0.0112	5.73	1.50–21.95	−0.07	0.9198	0.93	0.22–3.98
NLR								
High	1.34	0.0048	3.83	1.51–9.71	1.61	0.0133	5.00	1.41–17.75
PLR								
High	− 0.62	0.1787	0.54	0.22–1.32	0.94	0.0496	2.56	1.01–6.51

### Survival analysis

Median number of treatment cycles was 6 (range 1–12). Median PFS was 24 months and OS 69 months. Half of the patients (50%) were subjected to autologous hematopoietic stem cell transplantation (auto-HSCT). Median time to auto-HSCT was 12 months (range 1–36 months). Auto-HSCT was associated with significant extension of PFS (28 vs. 17 months; HR = 0.55, 95% CI 0.35–0.87, *p* = 0.0041) and OS (54 vs. 40 months; HR = 0.40, 95% CI 0.23–0.70, *p* = 0.0003).

**Fig. 4:**
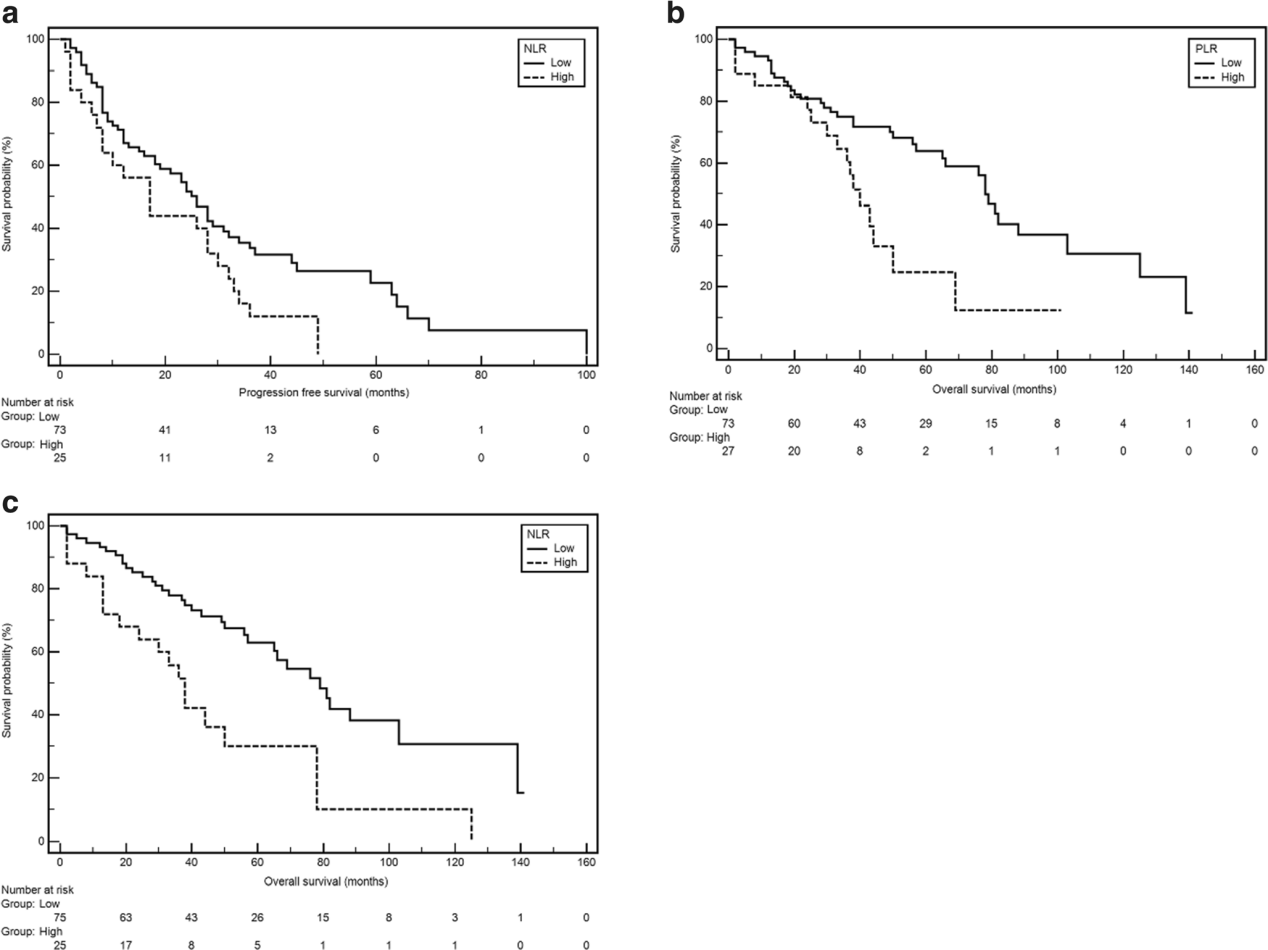
**a** Kaplan-Meier curves illustrating PFS differences between patients with low and high NLR. **b** Kaplan-Meier curves illustrating OS differences between patients with low and high NLR. **c** Kaplan-Meier curves illustrating OS differences between patients with low and high PLR

A significant longer median PFS was observed in patients with Durie-Salomon stage I or II (26 vs. 8 months; HR = 0.52, 95% CI 0.25–1.12, *p* = 0.0258). Similarly, longer PFS was noted in patients with normal renal function (A) (26 vs. 10 months; HR = 0.49, 95% CI 0.23–1.07, *p* = 0.0146) and normal ALB serum concentration (28 vs. 10 months; HR = 0.54, 95% CI 0.34–0.88, *p* = 0.0048). We also observed that patient with pre-treatment eGFR of more than 60 had about twice longer PFS compared to others patients (G3a/G3b/G4/G5D) (78 vs. 38 months, respectively; HR = 0.50, 95% CI 0.26–0.97; *p* = 0.0121) (Fig. 4a). On the other hand, shorter median PFS was observed in carriers of translocation (t4;14) (4 vs. 24 months; HR = 3.91, 95% CI 0.96–15.86, *p* = 0.0002) and interestingly in patients with higher NLR.

There was a significant difference in OS among patients with different staging. Patients in ISS stage I or II had significantly longer median OS compared to subjects with stage III (78 vs. 38 months; HR = 0.55, 95% CI 0.30–0.98; *p* = 0.0241). Among patients with normal renal function (“A” according to the Durie-Salmon scale), significant extension of OS was observed (78 vs. 33 months; HR = 0.42, 95% CI 0.16–1.14; *p* = 0.0126). Weight loss before treatment (defined as loss of at least 5% of body weight) was a significant factor associated with OS shortening (38 vs. 82 months; HR = 3.11, 95% CI 1.75–5.51; *p* < 0.0001). Male sex was associated with significantly higher risk of OS shortening (50 vs. 81 months; HR = 2.03, 95% CI 1.18–3.50; *p* = 0.0190). Among the analyzed cytogenetic abnormalities, the presence of translocation *t*(4;14) was associated with significant OS shortening (33 vs. 56 months; HR = 2.60, 95% CI 0.74–9.22; *p* = 0.0288). Significant OS extension was observed in patients with normal ALB level (78 vs. 38 months; HR = 0.57, 95% CI 0.32–1.01; *p* = 0.0381) as well as normal creatinine level (78 vs. 38 months; HR = 0.50, 95% CI 0.26–0.96; *p* = 0.0119). Interestingly, both high NLR and PLR were significantly associated with higher risk of OS shortening (38 vs. 79 months; HR = 2.45, 95% CI 1.22–4.49; *p* = 0.0010; Fig. 4b); 40 vs. 78 months; HR = 2.15, 95%CI 1.07–4.33; *p* = 0.0058; Fig. 4c; respectively). Results of univariate PFS and OS analysis were presented in Table 2.

Multivariate analysis using Cox’s regression model (Table 3) revealed factors which were independently associated with significantly higher risk of OS shortening: male sex (*p* = 0.0012; HR = 12.88, 95% CI 2.75–60.34), the presence of translocation (t4;14: *p* = 0.0218; HR = 3.84, 95% CI 1.22–12.07), weight loss before treatment (*p* = 0.0001; HR = 11.39, 95% CI 3.50–37.08), low ALB level (*p* = 0.0235; HR = 3.28, 95% CI 1.18–9.11), high NLR (*p* = 0.0133; HR = 5.00, 95% CI 1.41–17.75), and PLR (*p* = 0.0496; HR = 2.56, 95% CI 1.01–6.51). Factors which were independently associated with significantly higher risk of PFS shortening included presence of translocation t4;14 (*p* < 0.0001; HR = 15.18, 95% CI 4.98–46.26), low ALB level (*p* = 0.0021; HR = 3.47, 95% CI 1.58–7.64), higher serum concentration of creatinine (*p* = 0.0112; HR = 5.73, 95% CI 1.50–21.95), and high NLR (*p* = 0.0048; HR = 3.83, 95% CI 1.51–9.71).

## Discussion

A number of both experimental and clinical studies confirm the existence of a close relationship between chronic inflammation and malignancy, in virtually all of its stages: initiation, promotion, and progression [6–9]. Inflammatory cells capable of releasing a number of cytokines and growth factors cause damage to DNA, promote angiogenesis and lymphangiogenesis, and stimulate complex mechanisms of “escape” of malignant cells [10].

It has been reported that an increased number of lymphocytes infiltrating a tumor may be one of the markers of good prognosis [11–13]. In turn, the increase in the number of neutrophils or lymphopenia weakens the mechanisms of destruction of malignant cells, which promotes the formation of distant metastases [14]. Elevated values of neutrophils to lymphocytes ratio (NLR)—as an indicator of active infection—are associated with worse prognosis, weaker response to treatment, and shorter survival in patients with solid tumors [15].

Similar significance is attributed to the platelet to lymphocytes ratio (PLR)) [16, 17]. According to estimates, in up to 60% of patients with malignant tumors, thrombocythemia has a significant, unfavorable impact on the prognosis. Through complex mechanisms of hemostasis activation as well as cell signal transduction, an increased number of PLT stimulates cell proliferation and promotes metastasis [18, 19].

Bone marrow microenvironment and the way it interacts with myeloma cells are crucial in the pathogenesis of MM. The stromal environment determines the processes of growth, survival, migration, proliferation, and resistance to the treatment of neoplastically changed plasmocytes. Bone marrow stromal cells (BMSC), endothelial cells, and especially adhesion molecules on their surface have been shown to be critically important for the development of the disease. Their interactions through a series of proinflammatory cytokines released by BMSC and/or myeloma cells induce signaling pathways of proliferation and survival of monoclonal plasmocytes [20]. Therefore, all cells involved in the development of the so-called systemic inflammatory response could be important in the course of the disease.

There are only a few reports in the literature in which the NLR and PLR ratios were assessed in the context of prognosis or treatment efficacy in patients with MM.

Kelkitli et al. were the first to evaluate the value of NLR in patients with MM. The study included 151 patients and 151 healthy volunteers, appropriately selected for age and sex. NLR was significantly higher in myeloma patients than in the control group (2.79 ± 1.82 vs. 1.9 ± 0.61, respectively, *p* < 0.0001). It has been shown that NLR is an independent prognostic factor for OS and EFS (event-free survival) estimation. Patients with NLR < 2 at the time of diagnosis obtained longer OS compared to patients with NLR ≥ 2 (5-year OS were 87.5 and 42.4%, respectively, *p* < 0.0001). Similarly, longer EFS was observed in patients with NLR < 2 compared to patients with NLR ≥ 2 (5-year EFS rates were 88.4 and 41.8%, respectively, *p* < 0.0001) [21].

Onec et al. performed a retrospective analysis of 52 patients with MM. They showed that the NLR index depends on the concentration of CRP and β2-microglobin (*p* = 0.02, *p* = 0.001). They observed that patients with NLR > 1.72 were in a significantly higher stage of disease and had worse performance status and renal function. Median OS for the whole group was estimated at 35.1 months, and there was a significant difference in OS depending on the NLR (42.75 months for patients with NLR ≤ 1.72 and 26.14 months for patients with NLR > 1.72, *p* = 0.04) [22].

Romano et al. assessed the importance of the NLR index in relation to the efficacy of myeloma treatment with the use of immunomodulatory drugs (IMiDs) and proteasome inhibitors in various regimens with or without glucocorticoids. Three hundred nine patients were recruited for the study. Authors did not show a relationship between the NLR and the efficacy of single or double drug treatment regimens. However, they observed significant differences in the prognosis of patients treated with autologous bone marrow transplantation (ASCT). The median PFS was 22.1 in patients with NLR ≥ 2, versus 43.4 months in the NLR < 2 group (*p* = 0.017). In the group of patients with NLR ≥ 2 OS was 57.6 months, in the remaining subjects median OS was not reached (*p* = 0.002). Based on those findings, the authors proposed adding NLR to the ISS classification. They divided the subjects into three groups: very low risk—ISS1 and NLR < 2, very high risk—ISS3 and NLR ≥ 2, and the others were qualified for the standard risk group. They observed significant differences in the 5-year PFS depending on the ISS-NLR classification, respectively 39.3, 19.4, and 10.9% for the very low, standard, and very high risk groups. Five-year OS was 95.8, 50.9, and 23.6% for very low, standard, and very high risk patients according to ISS-NLR. Interestingly, the ISS classification itself was insufficient to differentiate patients against PFS and OS [23].

The results of two meta-analyses assessing the prognostic value of NLR in the course of the disease have also been published. Mu et al. analyzed 7 clinical trials involving a cohort of 1971 patients with myeloma and observed that elevated pre-treatment NLR was significantly associated with high stage of the disease according to the ISS (III vs. ISS I-II: OR 2427, 95% CI 1.268–4.467) as well as Durie-Salmon (III vs. I-II: OR 1.738, 95% CI 1.123–2.665) scales. Elevated NLR was associated with shorter OS (HR 2.084, 95% CI 1.341–3.238) and median PFS (HR 1.029, 95% CI 1.016–1.042). A linear relationship was found between increased NLR and mortality risk in patients with MM [24]. Zeng et al. analyzed the PubMed, Cochrane, and Embase databases. They studied data from eight clinical trials conducted jointly on a group of 1886 patients in 2013–2017. They reached the same conclusions—significantly shorter OS (HR 1.73, 95% CI 1.23–2.44, *p* = 0.002) and PFS (HR 1.74, 95% CI 1.11–2.73, *p* = 0.015) were noted in patients with NLR elevated prior to treatment. They also suggested that NLR correlates with the ISS stage of disease, the isotype of the disease, and the response to treatment [25].

The evaluation of the prognostic value of NLR and PLR in MM was also the subject of research by Li et al. Three hundred fifteen patients were randomized to receive regimens with bortezomib, thalidomide, and classic cytostatics. The cut-off points for the indicators were determined based on the analysis of the ROC curve—for NLR–2, for PLR–155. The authors confirmed that high NLR observed before treatment is an independent, unfavorable prognostic factor. However, they did not demonstrate a relationship between the PLR value, PFS, and OS time [26].

In a similar publication evaluating the importance of hematological indices of inflammation, Shi et al. confirmed the relationship between high NLR and MLR (monocyte-to-lymphocyte ratio) with unfavorable prognosis. In the case of PLR, they observed an inverse correlation; low values were associated with a shorter PFS: 32.3 vs. 40.4 months (*p* = 0.005) and OS 49.4 vs. 53.2 months (*p* = 0.008). The study was conducted in a group of 559 patients; the authors did not refer to the type of treatment used. Four (NLR), 100 (PLR), and 0.3 (MLR) were set as cut-off points [27].

The relationship between decreased PLR and prognosis in myeloma patients was confirmed in another study by Solmaz et al. Researchers recruited a cohort of 186 patients treated with chemotherapy containing vincristine/doxorubicine/dexamethasone (*n* = 100), cyclophosphamide/dexamethasone (*n* = 34), bortezomib/dexamethasone (*n* = 21), and others. They set following cut off points: 1.9 (NLR), 120.00 (PLR), and 0.27 (MLR) [28].

In contrast to the cited publications, in our study, we found a relationship between elevated PLR and shorter OS (40 vs. 78 months; HR = 2.15, 95% CI 1.07–4.33; *p* = 0.0058). Interestingly, high PLRs were observed in patients with low BMI and high serum CRP. In the current studies, patients treated with thalidomide-based regimen have not been isolated, which makes interpretation of the obtained results much more difficult.

## Conclusions

The results of our study confirm that simple, routinely available parameters, namely NLR and PLR, can be reliable, useful prognostic markers in estimating the survival of patients with MM. Furthermore, NLR can be a predictive marker for thalidomide based chemotherapy.

Results regarding the importance of NLR are mostly consistent with earlier observations. Differences observed with regard to PLR may result from the selection of the group in terms of the treatment used, and indicate the need to continue research on larger groups of subjects.
